# Cerebral hemodynamics during graded Valsalva maneuvers

**DOI:** 10.3389/fphys.2014.00349

**Published:** 2014-09-15

**Authors:** Blake G. Perry, James D. Cotter, Gaizka Mejuto, Toby Mündel, Samuel J. E. Lucas

**Affiliations:** ^1^School of Sport and Exercise, Massey UniversityPalmerston North, New Zealand; ^2^School of Physical Education, Sport and Exercise Sciences, University of OtagoDunedin, New Zealand; ^3^Laboratory of Sport Performance Analysis, Sport and Physical Education Department, Faculty of Sport Sciences, University of the Basque CountryVitoria-Gasteiz, Spain; ^4^Department of Physiology, University of OtagoDunedin, New Zealand; ^5^School of Sport, Exercise and Rehabilitation Sciences, College of Life and Environmental Sciences, University of BirminghamBirmingham, UK

**Keywords:** cerebral blood flow, hyperaemia, syncope, Valsalva maneuver, oxygenation

## Abstract

The Valsalva maneuver (VM) produces large and abrupt changes in mean arterial pressure (MAP) that challenge cerebral blood flow and oxygenation. We examined the effect of VM intensity on middle cerebral artery blood velocity (MCAv) and cortical oxygenation responses during (phases I–III) and following (phase IV) a VM. Healthy participants (*n* = 20 mean ± SD: 27 ± 7 years) completed 30 and 90% of their maximal VM mouth pressure for 10 s (order randomized) whilst standing. Beat-to-beat MCAv, cerebral oxygenation (NIRS) and MAP across the different phases of the VM are reported as the difference from standing baseline. There were significant interaction (phase ^*^ intensity) effects for MCAv, total oxygenation index (TOI) and MAP (all *P* < 0.01). MCAv decreased during phases II and III (*P* < 0.01), with the greatest decrease during phase III (−5 ± 8 and −19 ± 15 cm·s^−1^ for 30 and 90% VM, respectively). This pattern was also evident in TOI (phase III: −1 ± 1 and −5 ± 4%, both *P* < 0.05). Phase IV increased MCAv (22 ± 15 and 34 ± 23 cm·s^−1^), MAP (15 ± 14 and 24 ± 17 mm Hg) and TOI (5 ± 6 and 7 ± 5%) relative to baseline (all *P* < 0.05). Cerebral autoregulation, indexed, as the %MCAv/%MAP ratio, showed a phase effect only (*P* < 0.001), with the least regulation during phase IV (2.4 ± 3.0 and 3.2 ± 2.9). These data illustrate that an intense VM profoundly affects cerebral hemodynamics, with a reactive hyperemia occurring during phase IV following modest ischemia during phases II and III.

## Introduction

The Valsalva maneuver (VM) is commonly recruited during everyday activities such as lifting (Mac Dougall et al., 1992), defecation and coughing (Hamilton et al., 1944), and is characterized by changes in intrathoracic pressure that have a pronounced effect on venous return, cardiac output and blood pressures (Tiecks et al., 1995). Phase I of the VM is characterized by an increase in mean arterial blood pressure (MAP) at the onset of strain as the elevated intrathoracic pressure is translated to the arterial circulation; during phase IIa a reduction in atrial filling pressure decreases stroke volume with a baroreflex-mediated recovery in blood pressure, via an increased heart rate (phase IIb); phase III features a rapid decline in MAP as the strain is released, and; phase IV has a rapid recovery and overshoot of MAP as the now restored cardiac output is ejected into a constricted systemic vasculature (Goldberg et al., 1952; Tiecks et al., 1995; Pott et al., 2000).

The abrupt reduction in MAP during phase III challenges the regulation of cerebral perfusion and can result in syncope (Duvoisin, 1961) even after brief (10 s) VMs when standing (Perry et al., 2014). Syncope occurs due to an acute reduction in cerebral oxygenation leading to unconsciousness (Van Lieshout et al., 2003). The intrathoracic pressure perturbations during the VM are translated to the cerebrospinal fluid (Hamilton et al., 1944) such that increases in intracranial pressure (ICP) ensue (Greenfield et al., 1984), reducing transmural pressure in the cerebral arteries and thus flow (Haykowsky et al., 2003). Large changes in ICP potentially impair cerebral perfusion and have been used to induce occlusion (Gourley and Heistad, 1984). Whilst more intense VMs produce greater reductions in cerebral blood flow (CBF) velocity during Phase III (Perry et al., 2014), it is not known whether these fluctuations in flow coincide with changes in oxygenation.

Various tissues display reactive hyperemic responses, such as skeletal muscle and skin in response to exercise. The brain also displays reactive hyperemic flow following injury (Martin et al., 1997), stroke (Olsen et al., 1981) and surgical intervention (van Mook et al., 2005). Work in animals has shown that brief cerebral ischemia (5 s) can lead to a near-maximal hyperemic response (Gourley and Heistad, 1984). However, studies in healthy conscious humans exhibiting cerebral reactive hyperemia are scarce. Whilst a hyperemic response has been suggested during the phase IV response (Zhang et al., 2004), no concurrent beat-to-beat measures of CBF and oxygenation have been reported during a VM.

Therefore, the purpose of this investigation was to examine the cerebral hemodynamic response to graded VMs whilst standing. We hypothesized that the more intense VMs would result in greater reductions in both middle cerebral artery blood flow velocity (MCAv) and cortical oxygenation, and that the reduction in oxygenation would be matched by an increased flow velocity in phase IV of the VM in a dose-dependent manner.

## Materials and methods

Twenty healthy non-smoking male participants were recruited for the study (mean ± SD: age, 27 ± 7 years; body mass, 82 ± 17 kg; height, 176 ± 10 cm). Participants were informed of the potential risks and experimental procedures, and informed written consent was obtained. All procedures and protocols were approved by the University of Otago Human Ethics Committee and performed in accordance with the *Declaration of Helsinki*. All participants were free from disease and were not taking any medication. Participants abstained from strenuous exercise, alcohol and caffeine for at least 24 h before the experimental trial.

### Study design

Participants visited the laboratory on two occasions; one familiarization and one experimental trial. During the familiarization session the participants were familiarized with all experimental procedures and equipment, including practicing VMs at end-inspiration following a quiet period of spontaneous breathing. This enabled pre-VM hyperventilation to be minimized during experimental trials. Mouth pressure served as a surrogate for intrathoracic pressure (Mac Dougall et al., 1985; Morgan et al., 1993; Convertino et al., 2003; Heffernan et al., 2007) and reportedly reflects changes in esophageal pressure (Goldberg et al., 1952; Flemale et al., 1988). All VMs were performed in the standing position.

### Experimental protocol

During the experimental trial each participant first stood for 5 min, during which baseline measures were obtained, then completed a maximal VM for 10 s. Following recovery (i.e., when all values returned to baseline), relative VMs of 30 and 90% of the maximal Valsalva pressure were performed for 10 s, the order of which was randomized between participants. We have used these relative pressures previously to demonstrate graded cerebral blood flow velocity restriction (Perry et al., 2014). Visual feedback of the absolute mouth pressure was given in real time to aid the participant. Each VM was separated by 5 min or until values had returned to baseline. Participants were verbally instructed what pressure and duration to obtain, immediately before the performance.

### Measurements

Blood flow velocity in the right middle cerebral artery (MCAv) was measured using a 2-MHz pulsed Doppler ultrasound (DWL, Compumedics Ltd, Germany) using search techniques described elsewhere (Aaslid et al., 1982; Willie et al., 2011). The probe was secured with a plastic headband (DWL) to maintain insonation angle. Prefrontal cortical hemodynamics were obtained non-invasively (*n* = 10) using near infrared spectroscopy (NIRS, NIRO-200; Hamamatsu Photonics KK; Japan). Using NIRS, the concentration of oxygenated (O_2_Hb) and deoxygenated hemoglobin (HHb) as well as total hemoglobin (tHB) are obtained using the Modified Beer-Lambet law (Al-Rawi et al., 2001). Using these indices, total cortical oxygenation index (TOI% = O2Hb/tHb × 100) was calculated by the NIRS system (Spatially Resolved Spectroscopy method) from the light attenuation slope along the distance from the emitting point as detected by two photodiodes in the detection probe (emitter to detector distance was 4.5 cm). The probes were placed in an optically dense plastic holder to minimize extraneous light, and taped to the forehead (right side) with opaque tape.

Participants breathed through an adjustable mouthpiece, which allowed for the measurement of mouth pressure and the partial pressure of end-tidal CO_2_ (P_ET_CO_2_; gas analyser model ML206, ADInstruments, Australia). Mouth pressure was measured via a transducer attached to the mouthpiece and was used to measure the pressure during all VMs. Blood pressure was measured non-invasively and continuously using finger photoplethysmography (Finapres Medical Systems, Biomedical Instruments, The Netherlands), and heart rate was measured via three-lead electrocardiogram (ADInstruments). All data were acquired continuously via an analog-to-digital converter (PowerLab ML870; ADInstruments) at 1 kHz. Data were displayed in real time and recorded for off-line analysis using commercially-available software (v7.3.3 Lab Chart, ADInstruments).

Mean blood flow velocity (MCAv_mean_) and mean arterial blood pressure (MAP) were calculated as the integral for each cardiac cycle divided by the corresponding pulse interval. An index of cerebral vascular conductance (CVCi) was calculated via the equation MCAv_mean_/MAP.

### Data analyses

Baseline data were acquired in the last minute of each baseline period between VMs, and presented as the mean across that minute. All variables were attained at each of the four phases of the VM. This included the peak Phase I response, the average over the phase II, the nadir of phase III and the peak of phase IV. The short duration of the VM performed here did not lead to a clearly defined phase IIb response and as such all variables were averaged following the peak of the phase I response until the release of the VM, to represent phase II. Additionally, the area under the curve (AUC) for data during phase IV (from time when the variable exceeded, and subsequently declined back to, the pre VM value) was calculated to determine the total impulse of perturbation in accordance with the method described by Pruessner et al. (2003). To index the cerebral autoregulation response during Phases I and IV, the percentage change in MCAv_mean_ was divided by the percentage change in MAP from baseline or phase III, respectively, to assess differences in the MAP contribution to the MCAv_mean_ change. The Gosling pulsatility index for MCAv was calculated as (MCAv_systolic_− MCAv_diastolic_)/MCAv_mean_(Gosling and King, 1974).

Inferential statistical analyses of dependent variables were performed using a Two-Way ANOVA (phase × pressure). Data were assessed for approximation to a normal distribution and sphericity, with no corrections required. Main effects, AUC and time above baseline for MCAv were isolated using *post-hoc* pairwise comparisons (Bonferroni corrected, where necessary). Linear regression was used to determine the correlation between the phase dependent changes in MCAv, MAP, and TOI. All data were analyzed using SPSS statistical software (v20, Chicago, USA), with *a priori* statistical significance set at *P* ≤ 0.05. All data are presented as the mean ± SD absolute change from the baseline preceding the VM, unless stated otherwise.

## Results

**Figure 1:**
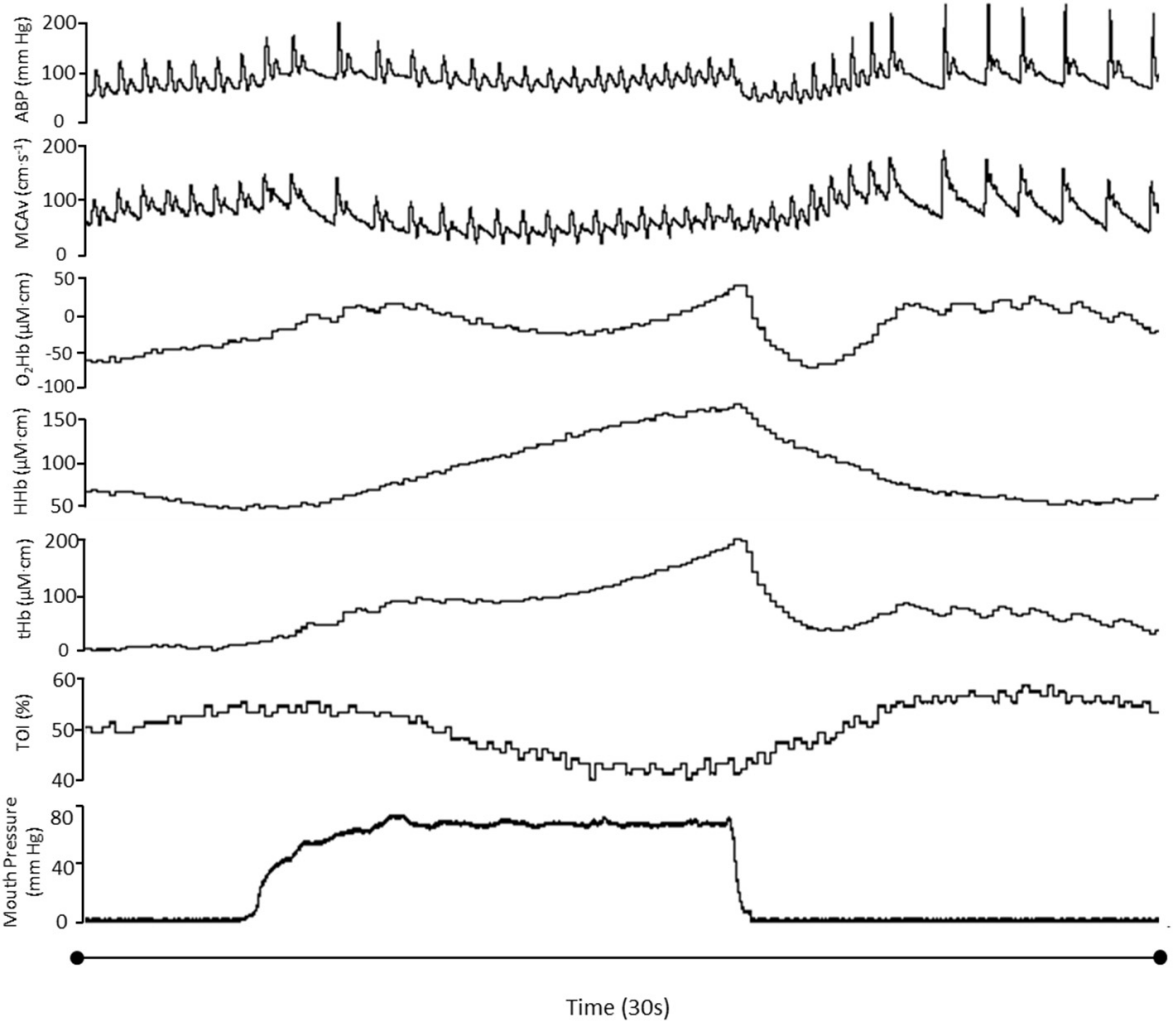
**Hemodynamic variables in one participant during a 90%VM**. ABP, Arterial blood pressure; MCAv_mean_, mean middle cerebral artery blood flow velocity; TOI, total oxygenation index; O_2_Hb, oxyhemoglobin; HHb, deoxyhemoglobin; tHb, total hemoglobin.

**Figure 2:**
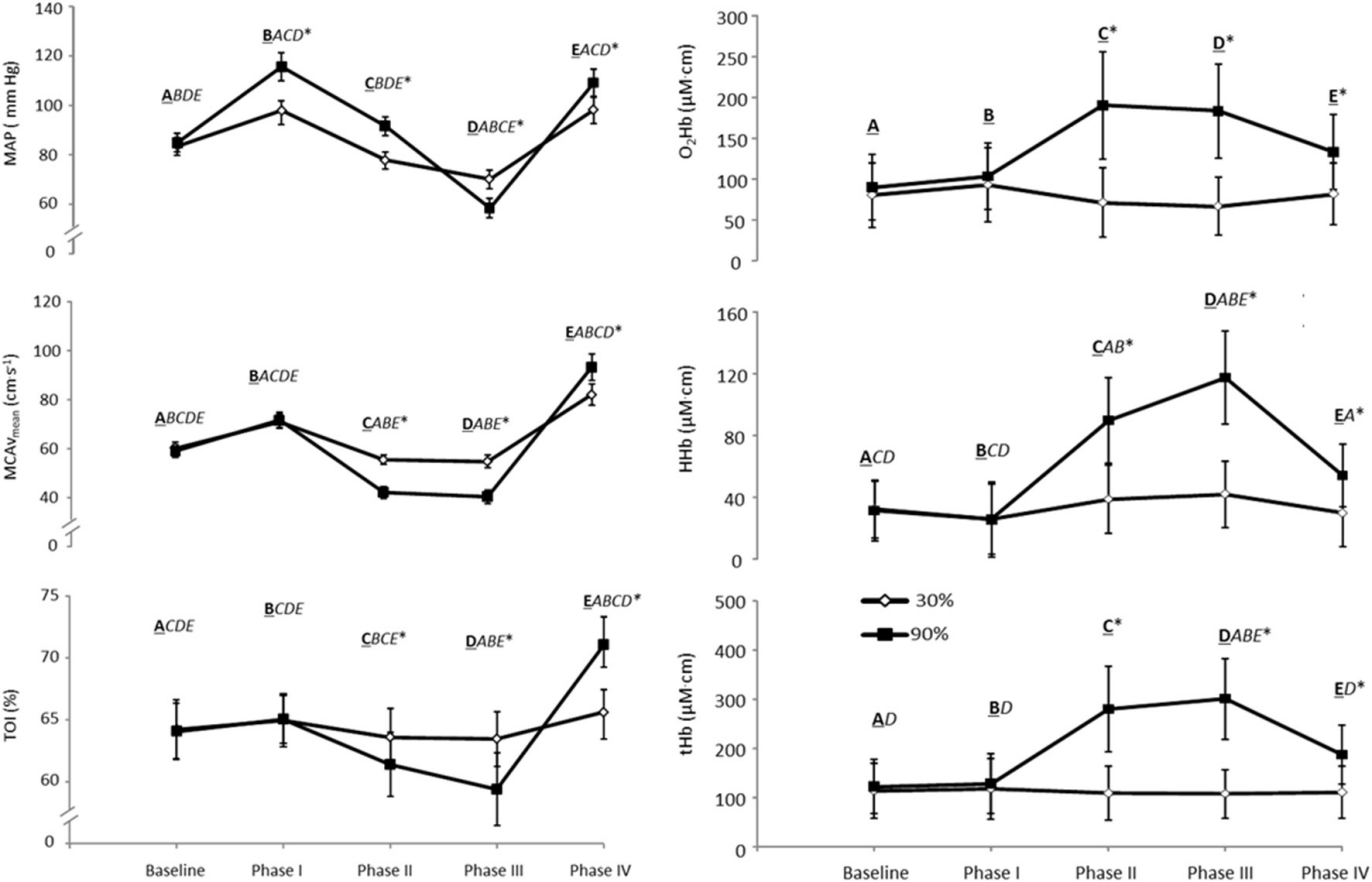
**MAP, Maximal mean arterial blood pressure; MCAv_mean_, Maximal mean middle cerebral artery blood flow velocity; TOI, Maximal total oxygenation index; O_2_Hb, Maximal oxyhemoglobin; HHb, Maximal deoxyhemoglobin; tHb, Maximal total hemoglobin (tHb, *n* = 10 for NIRS data) for each phase of the VM during a 30 and 90% relative intensity VM held for 10 s**. The bolded and underlined letters A–E represent the respective stages of the VM (baseline and phase I–IV on the x axis). The italicized letters represent differences between the phases (*P* < 0.05). ^*^Significant difference between 30 and 90%, *P* ≤ 0.05. Values are means ± SE.

A typical trace during a 90% VM is shown in Figure 1. Absolute changes from baseline during all phases of the VM are displayed in Table 1 and Figure 2. Mouth pressures were 24 ± 7 and 72 ± 21 for 30 and 90% VM respectively. Baseline data including P_ET_CO_2_ (grouped mean 33 ± 4 mm Hg) were unchanged between baseline periods. MAP, MCAv_mean_, TOI, O_2_Hb, HHb and tHb all demonstrated significant interaction effects (*P* < 0.01, Figure 2). Despite the greater increase in MAP during Phase I at 90% VM (*P* < 0.001, Figure 2), this did not correspond with greater increases in MCAv_mean_(*P* = 0.85). Phase III at 90% VM produced the lowest MCAv_mean_, MAP, and TOI values (Figure 2).

**Table 1:** **Change from baseline during all phases for 30 and 90% VM**.

			**Phase I**	**Phase II**	**Phase III**	**Phase IV**	**Pressure**	**Phase**	**Interaction**
Systolic MCAv, cm·s^−1^	98 ± 19	90	11 ± 12^**A***BCD*†^	−22 ± 15^**B**AD^^*^^†^	−23 ± 18^**C**AD^^*^^†^	40 ± 20^**D**ABC^^*^^†^	<0.001	0.06	<0.001
		30	9 ± 7	−8 ± 8	−11 ± 10	23 ± 18			
Diastolic MCAv, cm·s^−1^	41 ± 9	90	13 ± 10^**A***BCD*†^	−18 ± 14^**B**AD^^*^^†^	−19 ± 15^**C**AD^^*^^†^	27 ± 19^**D**BC^^*^^†^	<0.001	0.01	<0.001
		30	11 ± 7	−3 ± 6	−2 ± 8	19 ± 13			
CVCi, cm·s^−1^ mm Hg^−1^	0.74 ± 0.23	90	−0.04 ± 0.1^**A**BD^^*^	−0.3 ± 0.3^**B**ACD^^*^^†^	−0.1 ± 0.2^**C**BD^^*^	0.2 ± 0.1^**D***ABC*^^†^	<0.001	<0.001	<0.001
		30	0.02 ± 0.09	−0.02 ± 0.1	0.06 ± 0.09	0.2 ± 0.1			
PI	1.0 ± 0.1	90	0.8 ± 0.2^**A***BC*†^	1.3 ± 0.7^**B**AD^^*^	1.4 ± 0.7^**C**AE^^*^	0.8 ± 0.2^**D***BC*†^	<0.001	0.009	<0.001
		30	0.8 ± 0.01	1.0 ± 0.2	0.9 ± 0.2	0.8 ± 0.1			
Systolic BP, mm Hg	115 ± 17	90	32 ± 20^**A**BC^^*^^†^	−4 ± 21^**B***ACD*^	−29 ± 18^**C**ABD^^*^^†^	31 ± 26^**D**BC^^*^^†^	<0.001	<0.001	<0.001
		30	20 ± 17	−6 ± 9	−14 ± 12	23 ± 21			
Diastolic BP, mm Hg	68 ± 16	90	28 ± 16^**A**BC^^*^^†^	5 ± 16^**B**ACD^^*^	−20 ± 8^**C**ABD^^*^^†^	17 ± 14^**D**BC^^*^^†^	<0.001	<0.001	<0.001
		30	13 ± 8	−3 ± 7	−9 ± 6	12 ± 12			
HR, beats·min^−1^	74 ± 13	90	7 ± 15^**A***BC*^	19 ± 14^**B**ACD^^*^^†^	30 ± 16^**C**ABD^^*^^†^	6 ± 14^**D**BC^^*^	<0.001	<0.001	<0.001
		30	4 ± 10	8 ± 10	15 ± 10	−3 ± 14			

*Values are absolute mean ± SD change from baseline. VM, Valsalva maneuver; MCAv, middle cerebral artery velocity; CVCi, cerebrovascular conductance index; PI, Pulsatility index; BP, blood pressure; HR, heart rate; †, statistically different from baseline, P = 0.05*.

* *Statistically different from 30% within respective phase, P = 0.05. The bolded and underlined letters A–D represent the respective stages of the VM. The italicized letters represent differences between the phases (P < 0.05)*.

Peak MCAv_mean_ during phase IV was greater at 90% VM compared to 30% and phase I (both *P* < 0.05) despite a similar absolute MAP to that observed in phase I (*P* = 0.91) (Figure 2). Similarly, peak MCAv_mean_ during phase IV at 30% VM showed this higher flow velocity between phase I and IV (*P* < 0.05) despite similar MAP between the phases. TOI also showed these response profiles between VM intensities and between phases I and IV (Figure 2). The phase IV AUC for MCAv was not reliably affected by VM pressure (123 ± 73 and 257 ± 414 aU for 30 and 90% respectively; *P* = 0.17). Nor was time above baseline for MCAv (11 ± 6 and 12 ± 8 s for 30 and 90% respectively; *P* = 0.70).

The %MCAv/%MAP ratio demonstrated a significant effect of Phase only (*P* < 0.001), with a trend for an effect of VM intensity (*P* = 0.085). Specifically, the phase IV response showed the greatest ratio, of 2.4 ± 3.0 and 3.2 ± 2.9 for 30 and 90% VMs, respectively, which was significantly larger than the phase I response (0.8 ± 3.6 and 1.1 ± 1.3, *P* = 0.05). Time to peak for MCAv_mean_ during phase IV was 3.8 ± 1.8 and 4.4 ± 1.6 s for 30 and 90%, respectively (*P* = 0.34), and occurred before MAP (4.9 ± 2.0 and 6.4 ± 2.8 s, *P* = 0.007) and TOI (5.3 ± 1.4 and 8.0 ± 2.9 s, *P* = 0.008). Further, MCAv_mean_ had decreased from the Phase IV peak when TOI peaked (*P* = 0.02).

Finally, the decrease in MCAv_mean_ during phase III was not correlated with the increase during phase IV (*R*^2^ = 0.15; *P* = 0.2), nor did the decrease in TOI during phase III predict the increase in MCAv during phase IV (*R*^2^ = 0.07; *P* = 0.09).

## Discussion

The main novel findings of this study were that: (1) a more intense VM produced greater reductions in cortical prefrontal oxygenation during phases II and III; (2) the more intense VM resulted in higher peak MCAv_mean_ and TOI during phase IV; (3) during the phase IV response the %MCAv/%MAP ratio was above 2 for both mild and severe VMs—it was much greater than the phase I response and was indicative of a hyperemic response. Consistent with our hypothesis, the more intense VM produced greater reductions in flow velocity and oxygenation (TOI) during phases II and III and greater increases in both oxygenation and peak MCAv_mean_ during phase IV. However, the phase IV elevations in MCAv and TOI were unrelated to the preceding reductions in oxygenation and flow velocity at phase III.

Cerebral blood flow becomes restricted during and immediately following VMs, as evidenced by the reductions in MCAv_mean_ during phases II and III (Figure 2). The elevated venous pressure during a VM will increase cerebral blood volume (Pott et al., 2000; Gisolf et al., 2004), reduce venous outflow and contribute to the reduction in MCAv. Our NIRS measures of prefrontal oxygenation indicated that this reduction in flow (velocity) was sufficient to threaten cortical oxygenation, consistent with others measuring jugular oxygen tension during a VM (Meyer et al., 1966). This reduced flow, however, will be partially mitigated via an increase in arterial oxygen extraction (Trangmar et al., 2014). The continued increase in deoxygenated hemoglobin during phase II and III is consistent with this (HHb, see Figures 1, 2), resulting in an overall decrease in total oxygenation (TOI). We also observed an increase in total hemoglobin at phase III, which is indicative of increased cerebral blood volume; however, the pooling of blood in the venous system occurring at the site we measured seems unlikely, as it is more likely to occur in the larger veins and/or sinuses. One possibility is that this may reflect skin vascular pooling, since extracranial contamination may be present in these NIRS signals (Canova et al., 2011). However, the demand of extracranial tissue would not explain the large increase in HHb we observed, thus indicative of cerebral metabolism. More research is needed to clarify these contributions to the NIRS-derived parameters. Nevertheless, the concomitant reduction in blood flow and oxygenation (as indexed via Spatially Resolved Spectroscopy) can be sufficient to induce syncope during a VM, which has been reported as early as 3 s following the onset of the strain (Diehl et al., 2000). Indeed, some of our participants reported presyncope symptoms whilst performing the maximal and 90% VMs.

In response to cerebral occlusion, venous oxygen saturation has also been reported to increase substantially following occlusion (Symon et al., 1972; Gourley and Heistad, 1984). This mismatch between flow and demand has been termed “luxury perfusion” and has been reported following cerebral injury (Lassen, 1966) and neurosurgical procedures (van Mook et al., 2005). Although on a much shorter timescale than the aforementioned reports, our data are consistent with this luxury perfusion notion as evidenced by an observed temporal mismatch between peak reactive flow and oxygenation (~2–4 s). Gourley and Heistad (1984) have previously shown, in animals, that peak reactive cerebral hyperaemia is apparent after short periods of occlusion (5 s), with the duration of the hyperaemic flow dependent on the duration of ischemia. As both VMs were of the same duration in the current study, this may explain the similar AUC and duration for the phase IV MCAv_mean_ responses between relative pressures.

The brain demonstrates high-pass filter characteristics, with high frequency oscillations in MAP being translated to the cerebral circulation (Zhang et al., 1998). Whilst the increase in CBF during phase I may be restrained by the mechanical increase in ICP and subsequent reduction in transmural pressure (Haykowsky et al., 2003), the proportional increase in MCAv during phase IV was ~2- and ~3-fold greater than was explainable by MAP; (for 30 and 90%, respectively). In one participant who became syncopal, the peak phase IV response was associated with a near tripling of MCAv from baseline (no TOI measures available for this individual). A similar tripling of CBF has been reported in an animal-based study following an elevation of ICP above mean arterial pressure for 15 s (Gourley and Heistad, 1984). Thus, the increase in MCAv appears to be mediated by additional factors over-and-above that induced via the rapid rise in MAP, i.e., a reactive hyperaemia.

Although the more intense VM produced a greater reduction and subsequent overshoot in both TOI and MCAv during phase III and IV respectively, the two variables did not display a strong correlation. This may be due in part to the variable cerebral autoregulatory responses between individuals (Zhang et al., 2000), but is consistent with previous reports where a CBF:oxygenation mismatch was demonstrated during hyperaemic responses (Lassen, 1966). From our data it is difficult to ascertain the cause of the observed increase in CVCi during phase IV. A number of factors may have contributed to this response: (1) a residual vasodilation in response to the reduction in cerebral perfusion pressure during phases II and III as a result of the inherent latency of dynamic cerebral autoregulation (~5 s) (Zhang et al., 1998) (although for the given change in MAP when MCAv peak occurred, the proportionate contribution of MAP to this increase appears relatively small); (2) Following the release of the strain the sharp reduction in ICP and central venous pressure normalizes the transmural and arterial-venous pressures gradients respectively, aiding in flow restoration, and (3) The response is hyperemic in nature. When the MAP phase IV response is ablated the increase an MCAv is still apparent, which supports the notion of cerebral hyperemic flow (Zhang et al., 2004) even following VMs at moderate mouth pressures of 30 mm Hg, as we observed. Further, the VMs were performed in the standing position, which induces larger changes in MAP and MCAv (Pott et al., 2000; Perry et al., 2014). Therefore, it seems reasonable to assume that this response is, at least in part, hyperemic in nature, with peak reactive hyperemic flow velocity but not duration affected. Longer VMs may be required to induce longer hyperemic responses.

### Technological considerations

We used NIRS to provide a non-invasive measure of cerebral tissue oxygenation. The potential exists for extracranial contamination. Although NIRS strongly reflects cerebral oxygenation (Al-Rawi et al., 2001), the large perturbations in arterial blood pressure would produce some concomitant flow changes in extracranial vessels. During thigh cuff release, internal carotid artery flow recovers at the expense of external carotid artery flow and is a pertinent regulatory phenomenon to preserve CBF during acute hypotension (Ogoh et al., 2014a). The decrease in external carotid artery flow may reflect an arterial baroreflex mediated vasoconstriction (Ogoh et al., 2014a), which may falsely present as a decrease in cortical oxygenation (as measured using NIRS), despite an unchanged MCAv (Ogoh et al., 2014b). In contrast, in the current experiment both flow and oxygenation are in agreement, with changes in MCAv and TOI occurring concomitantly, indicative of a flow-mediated reduction in oxygenation in the brain parenchyma. Furthermore, the extracranial vessels are not subjected to changes in ICP. An abrupt decrease in intracranial CVCi, as demonstrated here during phase I, may shunt blood extracranially, maintaining flow in this vascular bed. As mentioned above, the steady rise in HHb from phase I to phase III seems likely to reflect the constant metabolic demand of the cerebral tissue (Figures 1, 2), which seems independent of the changes in O_2_Hb (and hence tHb) (Figure 2). While the initial rise in O_2_Hb during phase I may reflect the spike in BP and some extracranial shunting and associated increase in flow to the skin and face, the plateau and then late rise up until the strain release at phase III seems unlikely to be attributable to extracranial flow changes.

This study utilized blood flow velocity in the middle cerebral artery as a surrogate for global CBF. Changes in flow are adequately reflected by changes in flow velocity only when conduit artery diameter is unchanged (Valdueza et al., 1997), which appears to be true during moderate changes in MAP (Giller et al., 1993). Further, the retest reliability has been shown to be strong during repeated VMs using transcranial Doppler (Wallasch and Kropp, 2012). We have previously attempted to measure carotid artery diameter in order to clarify the effect of the VM on conduit artery diameter (Perry et al., 2014). However, the large changes in pressure within the jugular vein displace the internal and external carotid arteries making data acquisition within the time frame of the VM difficult. Therefore, the exact response of the conduit arteries is unknown with further examination required to establish blood distribution in both extracranial (face and skin) and intracranial circulations during the VM.

Alterations in arterial CO_2_ alter the efficacy of cerebral autoregulation (Aaslid et al., 1989). The P_ET_CO_2_, as a substitute for arterial PCO_2_, was unchanged between baselines, so cerebral tone would have been similar at the onset of the VM. The time course of the vascular response to changes in arterial CO_2_ is asymmetric with the “on” constant much slower than the “off” (Poulin et al., 1996). The time constant of the increase in MCAv during a step change in P_ET_CO_2_ is ~6 s (Poulin et al., 1996, 1998). Pott et al. (2000) reported that the reduction in arterial PCO_2_ contributed 10–15% to the reduction in MCAv during a 15-s VM. As VM duration in the current experiment was 10 s the influence of changes in arterial PCO_2_ would be expected to be less, although the exact effect of possible changes in arterial CO_2_ tension during the VM performed here is unknown. However, due to the delay in the vascular response and moderate changes in arterial PCO_2_ reported during longer VMs (Pott et al., 2000), the main driving factor during and initially following the VM appears to be the rapid changes in perfusion pressure rather than CO_2_.

## Conclusions

Performing a VM has dose-dependent effects on cerebral hemodynamics. Cerebral blood flow velocity and oxygenation decreases more during phase II and III of a 90% of maximum VM compared to a 30% effort, and is associated with a larger overshoot in flow velocity and oxygenation following the release of the strain (phase IV). Regardless of the magnitude of the reduction in flow and oxygenation, the subsequent overshoot was 2–3-fold greater than the increase in driving pressure (MAP), and was indicative of a reactive hyperaemia response.

## Funding

This study was funded by the Massey University School of Sport and Exercise.

## Author contributions

Blake G. Perry, James D. Cotter, Gaizka Mejuto, and Samuel J. E. Lucas were involved in conception and design of research. Blake G. Perry, James D. Cotter, Gaizka Mejuto and Samuel J. E. Lucas conducted experiment. All authors were involved in data analysis and interpretation. All authors edited and revised manuscript with all authors approving the final version of this article.

### Conflict of interest statement

The authors declare that the research was conducted in the absence of any commercial or financial relationships that could be construed as a potential conflict of interest.
